# Editorial: Crop abiotic stress: advances in germplasm/gene discovery and utilization

**DOI:** 10.3389/fpls.2024.1524430

**Published:** 2024-12-12

**Authors:** Guowei Li, Hui Song, Ran Hovav, Dayong Cui

**Affiliations:** ^1^ Institute of Crop Germplasm Resources, Shandong Academy of Agricultural Sciences, Jinan, China; ^2^ College of Grassland Science, Qingdao Agricultural University, Qingdao, China; ^3^ Institute of Plant Sciences, Agriculture Research Organization - The Volcani Center, Rishon LeZion, Israel; ^4^ Shandong Engineering Research Center of Rose Breeding Technology and Germplasm Innovation, School of Life Sciences, Qilu Normal University, Jinan, China

**Keywords:** editorial, crop, abiotic stress, advance, germplasm

Global climate variability exerts multiple abiotic stresses on crops, disrupting their growth and development, and resulting in substantial yield losses (Lobell and Gourdji, 2012). This alarming situation highlights the urgent need to explore the mechanisms through which plants mitigate these stressors (Lobell and Gourdji, 2012; Long et al., 2015; Song et al., 2024). Cultivated crops, despite their economic importance, often display limited genetic diversity, whereas their wild relatives, with higher genetic variability, exhibit stronger tolerance to both abiotic and biotic stresses (Fu, 2015; Salgotra and Chauhan, 2023). Thus, collecting, characterizing, and integrating both cultivated and wild germplasm has become a critical component of modern breeding programs. These diverse genetic resources offer significant opportunities for crop improvement but also pose challenges regarding their effective use (Fu, 2015; Liu et al., 2022; Salgotra and Chauhan, 2023). Developing core germplasm collections has therefore become a long-term strategy for optimizing the management and utilization of genetic resources.

The modern crop seed industry is evolving rapidly, but the current utilization of germplasm resources remains inadequate to meet its demands (Yan et al., 2023). A major bottleneck lies in the insufficient characterization of these resources, leading to limited access to high-quality germplasm with broad genetic diversity (Fu, 2015; Salgotra and Chauhan, 2023). Integrating germplasm exploration with investigations into plant responses to abiotic stresses will provide a robust framework for identifying superior materials and facilitating the development of breakthrough crop cultivars.

This Research Topic focuses on recent advances in germplasm and gene discovery related to abiotic stress management in crops, aiming to enhance our understanding of crop responses to abiotic stresses and promote the efficient utilization of genetic resources to support sustainable agricultural practices. Out of 30 submissions, 17 articles were accepted following rigorous peer review, including 16 research papers and 1 review. These studies cover various abiotic stresses—such as cadmium, calcium, CO_2_, cold, drought, heat, salt, selenium, waterlogging, and zinc—affecting crops such as peanut, rice, soybean, tobacco, and wheat. The findings offer valuable insights for exploring stress responses across diverse plant species.

## Studies on physiological and biochemical responses

Three articles examine physiological and biochemical responses to abiotic stresses. In soybean, foliar applications of amino acids and zinc not only maintained yield but also enhanced pod and branch numbers while promoting zinc biofortification (Han et al., 2024). Another study explored the unclear relationship between anthocyanin levels and salt stress in peanut, demonstrating that high anthocyanin content activates the antioxidant system, alleviating oxidative stress, and preserving photosynthetic efficiency under salt conditions (Li et al.). In another experiment, calcium-sensitive and calcium-tolerant peanut cultivars were compared under calcium-deficient conditions (Tang et al.). Calcium-sensitive cultivars exhibited a 22.75% reduction in yield, along with increased activities of antioxidant enzymes (SOD, POD, and CAT) and elevated MDA content (Tang et al.). In contrast, calcium-tolerant cultivars maintained stable yield and physiological performance, underscoring calcium’s essential role in crop productivity (Tang et al.).

## Studies on molecular responses

Several studies focused on molecular responses to abiotic stresses. In rice, sequencing of 541 cultivars followed by genome-wide association studies identified a candidate gene, *OsTMF*, as responsive to salt stress (Liu et al., 2024). Knockout experiments revealed that *OsTMF* promotes germination under salt conditions, demonstrating its potential utility for salt-tolerant breeding (Liu et al., 2024).

In wheat, researchers employed chromosome engineering strategies to introgress chromosome 7el1L from *Thinopyrum* species into wheat chromosome 7AL, producing recombinant lines with enhanced salt tolerance (Tounsi et al.). These lines exhibited notable physiological changes under salt stress, including increased photosynthetic pigment levels, accumulation of compatible solutes, and reduced antioxidant content (such as ascorbate) (Tounsi et al.).

In peanut, bioinformatics analysis identified 16 TPS (Trehalose-6-phosphate synthase) and 17 TPP (Trehalose-6-phosphate phosphatase) genes involved in cold stress responses (Zhong et al.). Notably, *AhTPS9* exhibited differential expression under cold treatment. Overexpression of *AhTPS9* in *Arabidopsis thaliana* improved cold tolerance by stabilizing the photosynthetic system and regulating sugar metabolism, making this gene a promising target for cold-tolerant peanut breeding (Zhong et al.).

In chickpea, Meta-QTL analysis revealed several genes involved in heat stress response, including pollen receptor-like kinase 3, flowering-promoting factor 1, and heat stress transcription factor A-5 (Kumar et al.). These genes influence flowering time, pollen germination, and overall plant development, offering valuable targets for heat-tolerant breeding programs (Kumar et al.).

In *Brassica juncea*, *BjNRAMP1* (Natural Resistance-Associated Macrophage Protein 1) was identified as a key gene involved in cadmium stress tolerance (Li et al.). Expressed in vascular tissues of roots, leaves, and flowers, *BjNRAMP1* facilitates cadmium and manganese accumulation when introduced into yeast and *Arabidopsis*, though its overexpression negatively affects plant growth (Li et al.).

A study in tobacco identified members of the Shaker K^+^ channel family, with *NtSKOR1B* up-regulated under salt stress (Yuan et al.). Mutants lacking *ntskor1* exhibited increased biomass and higher K^+^ content under salt stress, highlighting its potential role in improving salt tolerance (Yuan et al.). Another study used miRNA sequencing to explore drought stress responses in tobacco (Dai et al.). Thirteen miRNAs were differentially expressed under drought stress, including both known (e.g., nta-miR156b, nta-miR166a) and novel miRNAs (e.g., novel-nta-miR156-5p, novel-nta-miR209-5p) (Dai et al.). These miRNAs targeted genes involved in cell wall expansion, such as *EXT1* and *RWA2*, whose expression decreased under drought but recovered with selenium treatment (Dai et al.). A key regulatory pathway—novel-nta-miR97-5p-LRR-RLK-catechin—was identified, highlighting its importance in drought tolerance (Dai et al.).

In *Medicago sativa* (alfalfa), RNA-seq analysis of plants treated with methyl jasmonate (JA) and salt stress revealed two co-expression modules associated with antioxidant enzyme activity and ion homeostasis. Core genes identified included pyruvate decarboxylase and RNA demethylase, suggesting that JA enhances salt tolerance by modulating antioxidant responses and maintaining ion balance.

## Studies in non-crop plants

The Research Topic also includes studies on non-crop plants, offering insights applicable to crop improvement. For example, *Kandelia obovata* exhibits high tolerance to salt and waterlogging (Liu et al.). RNA-seq analysis identified 45 salt-responsive and 16 waterlogging-responsive genes involved in secondary metabolism, highlighting potential targets for enhancing abiotic stress tolerance in crops (Liu et al.).
